# Influence of Fishmeal-Free Diets on Microbial Communities in Atlantic Salmon (Salmo salar) Recirculation Aquaculture Systems

**DOI:** 10.1128/AEM.00902-16

**Published:** 2016-07-15

**Authors:** Victor Schmidt, Linda Amaral-Zettler, John Davidson, Steven Summerfelt, Christopher Good

**Affiliations:** a Department of Ecology and Evolutionary Biology, Brown University, Providence, Rhode Island, USA; b Josephine Bay Paul Center for Comparative Molecular Biology and Evolution, Marine Biological Laboratory, Woods Hole, Massachusetts, USA; c Department of Earth, Environmental and Planetary Sciences, Brown University, Providence, Rhode Island, USA; d The Conservation Fund's Freshwater Institute, Shepherdstown, West Virginia, USA; University of Michigan

## Abstract

Reliance on fishmeal as a primary protein source is among the chief economic and environmental concerns in aquaculture today. Fishmeal-based feeds often require harvest from wild fish stocks, placing pressure on natural ecosystems and causing price instability. Alternative diet formulations without the use of fishmeal provide a potential solution to this challenge. Although the impact of alternative diets on fish performance, intestinal inflammation, palatability, and gut microbiota has been a topic of recent interest, less is known about how alternative feeds impact the aquaculture environment as a whole. The recent focus on recirculating aquaculture systems (RAS) and the closed-containment approach to raising food fish highlights the need to maintain stable environmental and microbiological conditions within a farm environment. Microbial stability in RAS biofilters is particularly important, given its role in nutrient processing and water quality in these closed systems. If and how the impacts of alternative feeds on microbial communities in fish translate into changes to the biofilters are not known. We tested the influence of a fishmeal-free diet on the microbial communities in RAS water, biofilters, and salmon microbiomes using high-throughput 16S rRNA gene V6 hypervariable region amplicon sequencing. We grew Atlantic salmon (Salmo salar) to market size in six replicate RAS tanks, three with traditional fishmeal diets and three with alternative-protein, fishmeal-free diets. We sampled intestines and gills from market-ready adult fish, water, and biofilter medium in each corresponding RAS unit. Our results provide data on how fish diet influences the RAS environment and corroborate previous findings that diet has a clear influence on the microbiome structure of the salmon intestine, particularly within the order Lactobacillales (lactic acid bacteria). We conclude that the strong stability of taxa likely involved in water quality processing regardless of diet (e.g., Nitrospira) may further alleviate concerns regarding the use of alternative feeds in RAS operations.

**IMPORTANCE** The growth of the aquaculture industry has outpaced terrestrial livestock production and wild-capture fisheries for over 2 decades, currently producing nearly 50% of all seafood consumed globally. As wild-capture fisheries continue to decline, aquaculture's role in food production will grow, and it will produce an estimated 62% of all seafood consumed in 2020. A significant environmental concern of the industry is the reliance on fishmeal as a primary feed ingredient, as its production still requires harvest from wild fisheries. Our study adds to the growing body of literature on the feasibility of alternative, fishmeal-free diets. Specifically, we asked how fishmeal-free diets influence microbial communities in recirculating salmon farms. Unlike previous studies, we extended our investigation beyond the microbiome of the fish itself and asked how alterative diets influence microbial communities in water and critical biofilter habitats. We found no evidence for adverse effects of alternative diets on any microbial habitat within the farm.

## INTRODUCTION

The growth of the aquaculture industry has outpaced terrestrial livestock production and wild-capture fisheries for over 2 decades, currently producing nearly 50% of all seafood consumed globally (1). As wild-capture fisheries continue to decline, aquaculture's role in food production will grow, and it will produce an estimated 62% of all seafood consumed in 2020 (1, 2). Reliance on fishmeal as the traditional protein source in fish feed is among the chief economic and environmental concerns aquaculture faces today. Reliance on fishmeal drastically increases the environmental footprint of farmed fish, because fishmeal-based (FM) feeds require harvest from wild fish stocks, further straining marine ecosystems (3, 4). Fishmeal prices can fluctuate widely and have increased dramatically over the last decade. Research into fishmeal-free (FMF) diet formulations is therefore a priority for both conservationists and commercial farmers (5–9).

A number of carnivorous and omnivorous farmed fish species are capable of digesting poultry meals, nuts, soy, and grain on commercial scales (7, 10, 11), presenting the possibility that fishmeal can be eliminated as a component of fish feed. Previous studies have tested the feasibility of fishmeal-free feeds by examining how they impact different performance metrics, including growth (9, 12, 13), palatability (14, 15), nutrition (10), the fatty acid composition of the fillet (8, 16), and water quality (12). The majority of these studies found no differences across a range of metrics, indicating that alternative sources of protein are viable replacements for fishmeal.

However, open questions remain, and several studies have suggested that alternative diets may promote intestinal disorders and negatively impact conditions in the farm environment. For example, fish on alternative diets have higher excretion rates of ammonia and nitrate than those on diets including fishmeal and fish oil (14). Fish on alternative and traditional diets also exhibit differences in gut morphology and gut microbiota (6, 17–19). These findings suggest that additional research is required, both on the influence of alternative feeds on fish digestive systems and on the impacts of these diets on the critical microbial communities that maintain healthy farms.

Recently, interest in the use of closed-containment aquaculture with technologies of recirculating aquaculture systems (RAS) has grown, due to, among other things, the benefits associated with maintaining stable environmental and microbiological conditions within farm environments (20–22). These technologies are becoming increasingly popular, and the resulting seafood products demand a higher price in retail markets (23). However, microbial processes largely drive nutrient cycling and waste management, which are critical to water quality, fish health, and the success of these facilities. If and how the impacts of alternative feeds on the microbial communities in fish translate into changes to the microbial community of biofilters and the RAS farm environment as a whole has not been adequately assessed to date.

We tested the hypothesis that fishmeal-free diets influence the microbial communities in RAS water, biofilters, and salmon microbiomes using high-throughput 16S rRNA gene V6 hypervariable region amplicon sequencing. We raised postsmolt Atlantic salmon (Salmo salar) for 6 months in six replicate RAS tanks, three with traditional fishmeal diets and three with alternative fishmeal-free diets. At the end of the study, we sampled the intestines and gills of salmon (approximately 20 months posthatch), as well as the tank water and biofilters in each corresponding RAS unit. The findings presented in this paper demonstrate stability of biofilter microbial communities independent of diet but a consistent response of Lactobacillales in salmon intestines. Taken together, our results further highlight the feasibility of alternative-protein diets in aquaculture.

## MATERIALS AND METHODS

### Experimental RAS, research animals, and treatment descriptions.

All fish culture activities followed the Standard Operating Procedures for the Care and Use of Research Animals (Salmonid Fish) of The Conservation Fund Freshwater Institute (TCFFI), and all experimental protocols were approved by the TCFFI Institutional Animal Care and Use Committee (IACUC).

**FIG 1:**
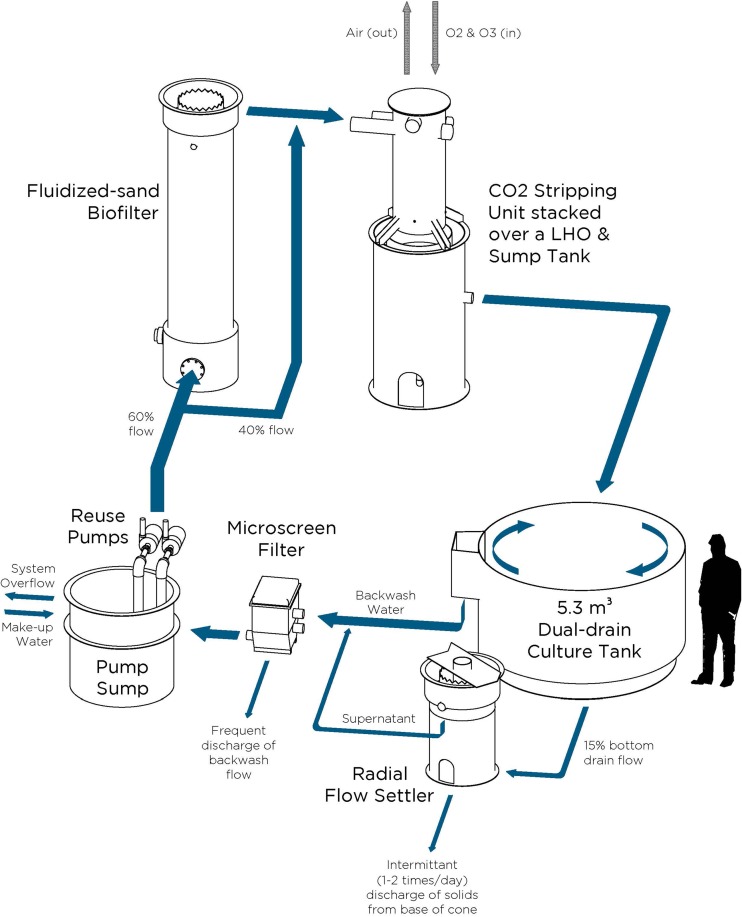
Process flow diagram for a single experimental RAS (9.5-m^3^ total system volume), illustrating the circular dual-drain culture tank, unit processes, movement of recirculating water, and location for makeup water addition. Our experiment consisted of 6 such units, three with fish fed traditional FM-based diets and three with fish fed alternative FMF diets.

Mixed-sex diploid Atlantic salmon, obtained as eyed eggs from a commercially available source, were hatched in a single incubation system and initially reared as fry in a flowthrough system. The fish were fed a standard commercial fishmeal-based diet during early rearing in the flowthrough system prior to transfer into six replicated RAS (Fig. 1). Each RAS (total water volume, 9.5 m^3^) consisted of a 5.3-m^3^ dual-drain culture tank, a radial-flow settler, a microscreen (60-μm) drum filter, a fluidized-sand biofilter, a carbon dioxide stripping column, and a low-head oxygenator (LHO). The total recirculation water flow was 380 liters/min (100 g/min). Aside from the addition of water (i.e., makeup water) to compensate for loss during daily radial-flow settler flushing, each RAS was operated at a 100% water recirculation rate, which provided an average system hydraulic retention time (HRT) of approximately 20 days. The average feed-loading rate was 3.2 ± 0.2 kg feed/m^3^ of daily makeup water; the tank HRT was approximately 15 min. A total of 220 fish (mean weight, 281 ± 5 g) were stocked in each RAS, for an initial stocking density of 12 kg/m^3^, and all the fish were allowed to acclimate for a period of 2 months. During this acclimation period, the fish were fed a standard commercial diet (43% protein, 24% fat), after which each RAS was randomly assigned one of two experimental diet treatments: either FM or FMF (the formulations are specified in Table 1).

**TABLE 1:** Nutritional contents of FMF and FM diets

Ingredient	Amt (g/kg)	
	FMF	FM
Mixed nut meal^*a*^	320	
Poultry meal^*b*^	295	160
Wheat flour^*c*^	99.4	195.1
Menhaden meal, mechanically extracted^*d*^		195
Fish oil, whitefish trimming oil^*e*^	182	
Fish oil, menhaden^*f*^		157.4
Soy protein concentrate^*g*^		128.5
Blood meal, spray dehydrated^*h*^		70.5
Canola oil		56.5
Corn protein concentrate^*i*^	35.6	
Dicalcium phosphate	32.5	
Monodicalcium phosphate		5
Vitamin premix^*j*^	10	10
Lysine-HCl	6.2	6.5
Choline Cl	6	6
Taurine	5	
dl-Methionine	2.8	4
Stay-C	3	2
Threonine	0.5	1.5
Trace mineral premix^*k*^	1	1
Astazanthin^*l*^	1	1

a Adaptive Bio-Resources; 540 g/kg protein.

b IDF Inc.; 759 g/kg protein.

c Manildra Milling; 120 g/kg protein.

d Omega Proteins, Menhanden Special Select; 628 g/kg protein.

e Bio-Oregon Proteins.

f Omega Proteins.

g Solae, Pro-Fine VF, 693 g/kg crude protein.

h ADF Inc.; 839 g/kg protein.

i Cargill, Empyreal 75; 761.0 g/kg protein.

j ARS 702. Contributed (per kg diet): vitamin A, 9,650 IU; vitamin D, 6,600 IU; vitamin E, 132 IU; vitamin K_3_, 1.1 g: thiamine mononitrate, 9.1 mg; riboflavin, 9.6 mg; pyridoxine hydrochloride, 13.7 mg; pantothenate dl-calcium, 46.5 mg; cyanocobalamin, 0.03 mg; nicotinic acid, 21.8 mg; biotin, 0.34 mg; folic acid, 2.5 mg; inositol, 600 mg.

k ARS 640. Contributed (mg/kg diet); zinc, 40; manganese, 13; iodine, 5; copper, 9.

l DSM Nutritional Products.

These diets were manufactured at the U.S. Department of Agriculture (USDA) Agricultural Research Service (ARS) Fish Technology Center (Bozeman, MT, USA); the primary protein sources in the FMF diet were mixed nut meal and poultry meal. Automated feeders provided the fish with these diets every 2 h around the clock while maintaining a full 24-hour photoperiod. Over the study period, the feeding rate for each RAS was adjusted separately based on observations of fish feeding activity and wasted feed. On a monthly basis, fish length and weight data were collected from a random sample of 60 to 90 fish per RAS, and daily mortalities were recorded. Weight, water temperature, and mortality data were used to calculate thermal growth coefficients (TGC) and cumulative survival. Specifically, the TGC was calculated as follows: TGC = [(end weight^1/3^ − start weight^1/3^)/(number of days between × average temperature)] × 1,000.

Throughout the 6-month study, a range of water quality and RAS unit process data were collected; the methodologies and frequencies of testing for each parameter measured are summarized in Table S1 in the supplemental material. The culture tank water was sampled weekly and evaluated on site according to methods outlined in Table S1.

### Microbiome sample collection.

At the end of the experimental period, we sampled four microbial habitats: (i) intestinal tracts, (ii) gill tissue, (iii) RAS water, and (iv) biofilter medium (i.e., the substrate upon which the nitrifying bacteria exist in biofilms; in this case, the medium was fluidized sand). For fish tissue sampling, we randomly sampled 6 fish from each RAS via dip net collection; euthanized them with an overdose (200 mg/liter) of tricaine methanesulfonate (MS-222; Western Chemical, Inc., Ferndale, WA, USA); and sequentially placed them, right side down, on a repeatedly spray-disinfected (95% ethanol) surface for dissection. The exterior of each fish was first spray disinfected; then, using sterilized instruments, the left operculum was removed for ease of access to the underlying gill tissue. The instruments were resterilized with an ethanol dip and flaming, followed by careful removal of the second gill arches, which were placed into dry 15-ml sterile cryogenic storage vials (Thermo Fisher Scientific, Rochester, NY, USA) and frozen at −20°C within 1 h. We then sprayed each fish with alcohol, resterilized the instruments, and carefully incised the ventral midline to avoid puncture of the digestive tract. Tissue sections consisted of a 4- to 5-cm section of the midintestine, tissue and contents combined, aseptically removed and placed in sterile cryogenic storage vials. Biofilter medium samples were collected in cryogenic vials using a sampling port located at approximately the midheight of the biofilters, after first opening the ports and allowing flow for approximately 20 s prior to sample collection. Water samples were collected at the sidewall box sampling port of each culture tank by filtering 1 liter of water through 0.22-μm Sterivex (Millipore, Billerica, MA) filters. All the samples (tissues, biofilter medium, and water) were immediately placed on dry ice and shipped overnight to the Marine Biological Laboratory (MBL) in Woods Hole, MA.

### DNA extraction.

We extracted intestine samples by first isolating a 0.5-g piece of the midintestine using sterile tools. Any material connected to the outside of the intestine was removed, including fat or muscle, but fecal material contained inside the intestine was not removed. We prepared gill tissue in a similar fashion, although these samples required less removal of auxiliary material. We added the intestine or gill tissue directly to MoBio (Carlsbad, CA) PowerBiofilm bead tubes and conducted extractions according to the manufacturer's protocol. Biofilter samples were extracted using a similar method, except that 0.5 g of biofilter sand was first centrifuged to remove the water before being added to the PowerBiofilm bead tube.

We extracted Sterivex filters containing water samples as described previously (24). Briefly, the filters were removed from cartridges and added to 1.5 ml of PureGene (Valencia, CA) cell lysis solution with 4 μl PureGene lytic enzyme and incubated for 30 min. MoBio DNA extraction beads (MoBio catalog number 13113-50) were then added, and the entire solution was vortexed on high using a MoBio VortexGenie adaptor for 10 min. The solution was heated to 80°C for 5 min and revortexed briefly, followed by protein and final DNA precipitation.

### Sequencing and bioinformatics.

We sequenced the V6 hypervariable region of the bacterial 16S rRNA gene using a custom 2-step fusion primer PCR amplification. First, we performed an initial 20-cycle PCR in triplicate using a cocktail of standard forward and reverse universal bacterial primers (967F and 1064R). We then amplified the product in a second, 10-cycle PCR using primers with Illumina HiSeq adaptors and barcodes attached to their 5′ ends. Our fusion PCR protocols and primer sequences are further explained elsewhere (25; https://vamps.mbl.edu/resources/primers.php). Paired-end sequencing was conducted at the MBL Keck sequencing facility on an Illumina HiSeq 1000 and generated 100-bp reads with 100% overlap of forward and reverse sequences. The forward and reverse sequences were aligned, and quality filtering removed any read with a mismatch between the forward and reverse alignments, following standard Illumina paired-end-sequencing protocols (25). In order to detect contamination, a negative control was also sequenced. Five reads were found to be highly abundant in our negative control. These reads contained no close relatives in our data set and were found across negative controls from other data sets sequenced at the same facility. As such, they were deemed contaminants and removed from our data set prior to bioinformatics analysis. Their sequences are included in Data Set S1 in the supplemental material.

After quality filtering and contaminant removal, operational taxonomic units (OTUs) were created using minimum-entropy decomposition (MED) to cluster sequences into MED OTUs (26). We used an MED-calculated minimum-substantive-abundance threshold to remove any OTU with fewer than 1,895 reads in its most abundant unique sequence and more than 1 nucleotide of variation across all sequences in the OTU. MED determines locations in marker sequences that contain discriminating information by determining each location's entropy across all samples in a study. It then partitions sequences into increasingly higher-resolution clusters based on these high-entropy locations until no additional meaningful sequence variation is found within a cluster (i.e., minimum entropy has been attained). By using only these high-entropy locations within a marker gene, the method is able to cluster sequences using only biologically meaningful sequence variation as opposed to traditional distance-based clustering methods (e.g., UClust), which use the entire sequence and are more susceptible to nonbiological noise (e.g., sequencing error). As a result, the method provides more ecologically meaningful results than traditional clustering and has been demonstrated across a wide and increasingly diverse range of microbial habitats (27–30). Our full MED analysis is publically available on figshare (https://dx.doi.org/10.6084/m9.figshare.2061078.v1) and can be navigated using the Index.html file after decompression of the folder.

After decomposition was complete, we used the most common sequence in a given OTU as a representative sequence for that OTU and the GAST pipeline (31, 32) to assign taxonomy to each representative sequence. Finally, we uploaded our resulting MED matrix to the Visualization and Analysis of Microbial Population Structure (VAMPS) interface and normalized the entire matrix to both total (relative abundance) and maximum. Further public analysis and exploration of these data are possible on the VAMPS website (https://vamps.mbl.edu/) under the data set name Schmidtetal_AEM_OTUs in the community visualization tab.

### Statistical analyses.

Fish performance and water quality data were analyzed using restricted maximum likelihood (REML) mixed models, with tank assigned as a random effect and time included as a random covariate in the models. These statistical analyses were carried out using SYSTAT 13 (Systat Software Inc., San Jose, CA, USA).

To test for community level differences between treatments, we used the permutation-based multivariate analysis of similarity (ANOSIM). First, we compared microbial communities across the four habitat types, regardless of diet, using a single-factor ANOSIM with 9,999 permutations on a Bray-Curtis similarity matrix as implemented in PrimerE v6.1. Pairwise ANOSIMs were then run on all possible combinations of habitats to determine where significant differences occurred. We then tested for the influence of diet and tank membership for each habitat type individually, using a nested ANOSIM where tank was nested within diet. Alpha values were corrected for multiple comparisons in all cases.

We also ran hierarchical cluster analysis on our Bray-Curtis similarity matrix across all samples, as implemented in VAMPS (33). Observation of these analyses revealed an independent cluster of eight intestine samples with a single genus that had >50% relative abundance and clustered independently of tank or diet treatment. Because these samples had such a high abundance of a single genus (and often a single OTU with >80% relative abundance), we reran the above-mentioned ANOSIM analyses without these eight outlier samples.

To determine which MED OTUs were driving differences between diets or tanks, we used similarity percentage (SIMPER) analysis, again implemented in PrimerE v6.1. We visualized the results using nonmetric multidimentional scaling (NMDS) plots of Bray-Curtis resemblance matrices and covariance ellipsoids drawn using the betadisper{vegan} function in R (34), as implemented in the oligotyping pipeline (27). Based on the results of our SIMPER analyses, we also tested individual taxa for significant differences between treatments using pairwise Student *t* tests and Bonferroni multiple-comparison corrections.

We also tested if tank water and intestinal communities within a tank were more similar to each other than tank water and intestinal communities in different tanks. This analysis allowed us to determine if tank water communities had any influence on intestine communities. To do this, we compared mean within-tank Bray-Curtis similarities to between-tank comparisons for the two habitats. Significance between means was calculated using Student *t* tests. We note here that water and tissue genomic DNAs were extracted using different extraction protocols, so differences in these microbial communities may be due in part to biases of each protocol.

Alpha diversity values were calculated in VAMPS (33) using Shannon diversity and observed species after all samples were rarified to 25,000 sequences. Because subsampling can yield somewhat different results across repeated iterations, each diversity metric was measured five times with independent subsampling, and the mean value was used. Alpha diversities between habitats were compared using pairwise *t* tests with Bonferroni corrections for multiple comparisons.

### Accession number(s).

All original sequence files and minimum information about a marker gene sequence (MIMARKS)-compliant data (35) (see Data Set S2 in the supplemental material) have been deposited in NCBI's Sequence Read Archive (SRA) under BioProject accession number PRJNA302804.

## RESULTS

### Water quality and salmon performance.

Among the water quality parameters assessed, nitrate-nitrogen and total-ammonia nitrogen were significantly higher in FMF RAS, while total phosphorus, total suspended solids, and true color were significantly higher in FM RAS (Table 2). Despite these differences, the water quality parameters were all within established safe ranges for salmonids. No significant differences were found in salmon performance (growth rate, final weight, and survival) between our diet treatments (Table 2; the raw data values are included in Data Set S3 in the supplemental material).

**TABLE 2:** Influence of diet type on salmon growth and survival and water quality parameters

Parameter^*c*^	Value^*a*^	
	FMF diet	FM diet
Water quality		
Alkalinity	206 ± 2	208 ± 2
Carbon dioxide	4 ± 0	3 ± 0
cBOD	0.9 ± 0.1	0.9 ± 0.1
Dissolved oxygen	10.0 ± 0.0	10.0 ± 0.0
Heterotroph bacteria (CFU/ml)	437 ± 83	493 ± 121
Nitrite nitrogen	0.05 ± 0.04	0.03 ± 0.02
Nitrate nitrogen^*b*^	65 ± 2	57 ± 1
Oxidative reduction potential (mV)	248 ± 1	255 ± 4
pH	8.1 ± 0.0	8.1 ± 0.0
Temperature (^o^C)	15.2 ± 0.0	15.2 ± 0.0
Total-ammonia nitrogen^*b*^	0.17 ± 0.01	0.13 ± 0.01
Total nitrogen	54 ± 1	49 ± 1
Total phosphorus^*b*^	4.3 ± 0.1	0.9 ± 0.0
Total suspended solids^*b*^	1.3 ± 0.2	1.7 ± 0.1
True color (Pt-Co units)^*b*^	20 ± 2	25 ± 2
UV transmittance (%)	81 ± 1	79 ± 1
Salmon performance		
Thermal growth coefficient	2.14 ± 0.05	2.12 ± 0.01
Overall survival (%)	99.7 ± 0.3	99.8 ± 0.2
Size at end of study (kg)	1.75 ± 0.076	1.720 ± 0.065

a In milligrams per liter unless otherwise indicated.

b Significant difference.

c cBOD, carbonaceous biochemical oxygen demand.

### Microbial community structure.

Sequencing of the V6 hypervariable region of the bacterial 16S rRNA gene yielded a total of 9,473,000 reads after filtering for sequence quality and contamination across 63 samples (mean ± standard error [SE], 150,398 ± 14,612). MED analysis removed 1,510,650 reads due to minimum-substantive-abundance filters, resulting in 7.9 million reads represented after all quality filtering (Table 3 ). Sequencing depth varied according to the microbial habitat within the RAS, with biofilter and water habitats showing higher average sequencing depth than gills and intestine; however, we note that samples were rarified to median depth prior to any analyses, eliminating potential biases due to sequencing depth (26).

**TABLE 3:** Sample breakdown, MED analysis, and ANOSIM results^*a*^

Parameter	Value
Sample breakdown [mean no. of sequences (SE)]	
Biofilter (18 samples)	231,264 (53,434)
Gills (10 samples)	63,354 (20,034)
Intestine (26 samples)	66,171 (12,735)
Water (6 samples)	207,981 (78,609)
MED analyses	
No. of sequences analyzed	9,475,043
No. of sequences represented after quality filtering	7,964,393
No. of raw nodes (OTUs) (before the refinement)	494
No. of final nodes (OTUs) (after the refinement)	495
Nested ANOSIM tests^*b*^	
Tank effect	
Biofilter	<0.001^*c*^
Gills	NS^*d*^
Intestine	0.034^*c*^
Water	NS
Diet effect	
Biofilter	0.1
Gills	NS
Intestine	0.029 (0.0002)^*c*^
Water	NS

a Number of samples for each RAS habitat, and mean sequencing depth are shown, along with the results of MED clustering analysis (for details, see http://merenlab.org/2014/11/04/med/).

b Results from multivariate statistical analysis. The number in parentheses is the significance of intestinal groupings by diet after removal of outlier samples.

c Statistically significant result.

d NS, not significant.

Richness and diversity varied significantly between habitats, with intestine and gill samples containing significantly fewer observed OTUs than biofilter and water samples (all pairwise *t* tests had *P* values of <0.0125) (see Fig. S1 in the supplemental material). Within habitat types (i.e., gill, intestine, water, and biofilter), diet treatment influenced only biofilters, where FMF diets had slightly yet significantly greater richness and Shannon diversity than FM diets (richness, FMF = 346 ± 3.6, FM = 316 ± 4.6) (see Fig. S1 in the supplemental material). Neither richness nor Shannon diversity varied significantly within any other habitat type according to diet. We note that although the gill samples showed a large mean effect size, low replication and large variance likely led to nonsignificant differences.

### OTU level analyses.

**FIG 2:**
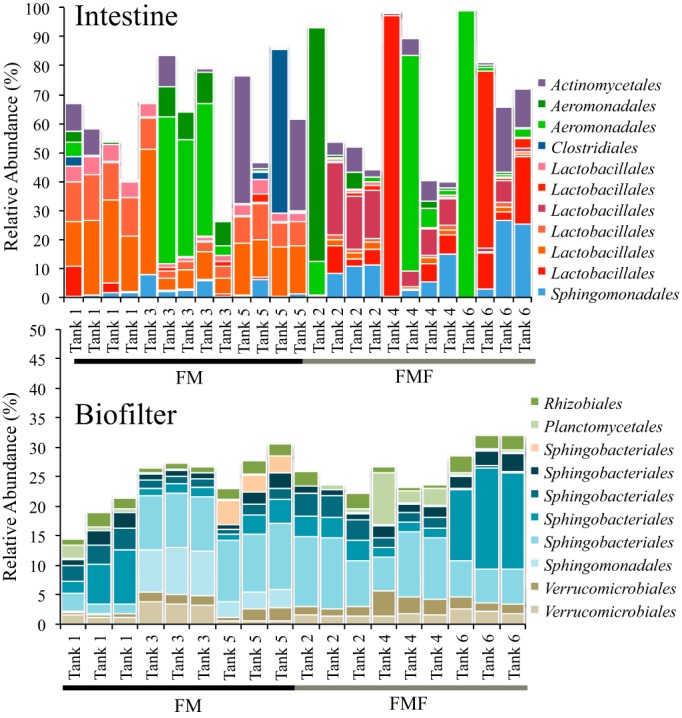
Relative abundances of the top 11 most abundant MED OTUs across intestine samples (top) and the top 10 most abundant MED OTUs across biofilter habitats (bottom) by tank and fish type.

Habitats varied significantly in their compositions. The most abundant OTUs in biofilter habitats belonged to the orders Sphingobacteriales and Nitrospirales, while intestine habitats were dominated by Lactobacillales and Aeromonadales OTUs (Fig. 2; see Fig. S2 in the supplemental material). Burkholderiales, Neisseriales, and Nitrospirales OTUs dominated water habitats. Gills showed the highest variation across all habitats but were also the only habitat to show a dominance of Enterobacteriales OTUs (see Fig. S2 in the supplemental material). Very few OTUs were highly abundant in more than a single habitat, and no OTU occurred among the top 10 most abundant OTUs in more than 2 habitats (see Fig. S2 in the supplemental material). Gills shared several highly abundant OTUs with both water and intestine samples, while biofilters shared only a single top 10 OTU with another habitat (Sphingomonas OTU 4011 occurred in both water and biofilter samples at high abundance).

**FIG 3:**
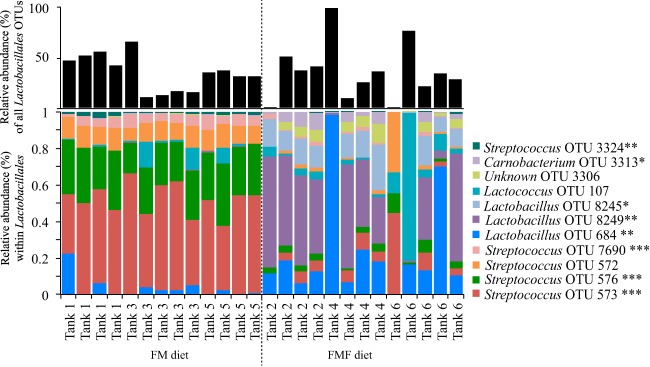
Proportion of all Lactobacillales represented by a given Lactobacillales OTU across all intestinal samples (bottom) and the total relative abundances of all Lactobacillales OTUs in the community (top). The asterisks show the significance level of Student's *t* test for each OTU between FM and FMF treatments (*, *P* < 0.004; **, *P* < 0.0004; ***, *P* < 0.00004).

Large variability existed for some highly abundant OTUs across a single habitat. For example, a single Lactobacillales OTU (Lactococcus OTU 107) represented 96% of the intestinal microbiome of a single fish yet averaged only 3.5% relative abundance in all other fish (Fig. 2 and 3; see Fig. S2 in the supplemental material). The extremely high abundance of this Lactococcus OTU was confirmed by repeated sequencing of the original genomic DNA, suggesting it was not an artifact or PCR bias. Similarly, a single Aeromonadales OTU (Aeromonas OTU 3544) represented 81% of the intestinal microbiome in a single fish but averaged only 2.3% across all other intestinal samples. Another Aeromonadales OTU (Aeromonas OTU 3545) occurred across five fish samples from different tanks and diets at very high abundance (>45% relative abundance) but was rare across all other fish. The influence of these highly abundant Aeromonadales OTUs is evident from intestinal samples that group together in hierarchical cluster analysis, all with a single highly abundant Aeromonas OTU (see Fig. 6). Interestingly, these highly abundant OTUs did not influence the communities of other habitats within the same fish or tank. For example, the Lactobacillales OTU found at 96% in an intestine sample from tank 4 was found at only 0.6% in the gill sample from the same fish and was not found at all in the biofilter or water from that tank.

Previous research has suggested fishmeal-free diets have a particular influence on Lactobacillales in the microbiomes of salmonids (6). Our study confirmed that OTUs within the order were highly variable between the two treatments (Fig. 3) and appeared to be driven by diet. In particular, Streptococcus OTUs 573, 576, and 572 had significantly greater relative abundance in FM treatments versus FMF treatments, while the opposite was true for Lactobacillus OTUs 684, 8245, and 8249 (Fig. 3). Interestingly, although we observed shifts at the OTU and genus levels, the differences all occurred within the Lactobacillales, which dominated the microbial community in most samples independently of diet (Fig. 2 and 3). We note that none of these OTUs are significantly different when compared across tanks within the same feed treatment, suggesting there is no evidence for a tank effect of the taxa (Fig. 3).

The biofilter habitat was less variable in general (Fig. 2), although a similar pattern occurred, in which a single OTU would be abundant in some tanks and absent from others. This pattern is evident with a Flavobacteriales OTU (Flavobacterium OTU 1366) that occurred only in tanks 1 and 4 above 1% relative abundance, a Shingobacteriales OTU (Haliscomenobacter OTU 5418) that occurred only in tank 5 above 1% relative abundance, and a Planctomycetales OTU (Rhodopirellula OTU 52) that occurred only in tank 5 above 1% relative abundance (Fig. 2). The variation in relative abundance that these OTUs show between tanks partly drove the strong within-tank clustering of biofilter microbial communities (see Fig. 6 and Fig. S3 in the supplemental material).

### Community level analyses.

**FIG 4:**
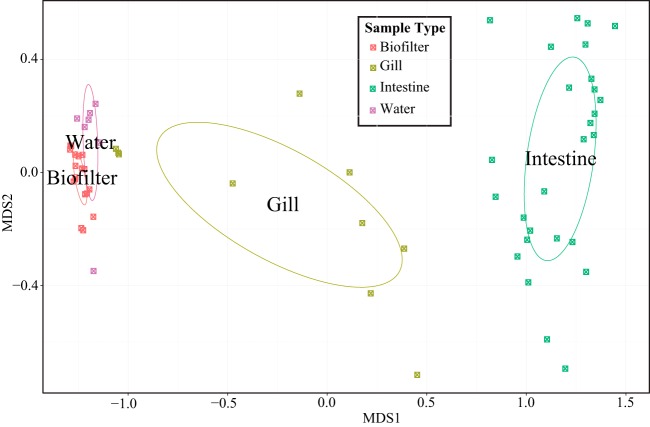
MDS plot of the MED similarity matrix between all study samples. The samples are colored according to type. The circles represent covariance ellipsoids for each sample group (a measure of variance within the sample). Note that diets are not distinguished.

**FIG 5:**
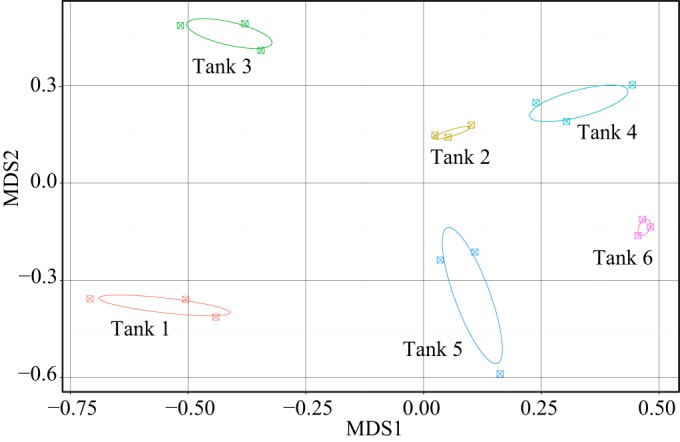
MDS plot of the MED similarity matrix between biofilter samples. The samples are colored according to tank. The circles represent covariance ellipsoids for each tank (a measure of variance within the sample). Tanks 1, 3, and 5 are FM diets, while 2, 4, and 6 are FMF.

Statistical comparisons of bacterial communities across habitat types identified differences between habitats (e.g., biofilter versus intestine) and within habitats (e.g., FM versus FMF intestine samples). All possible pairwise ANOSIM tests between habitats showed significance at a Bonferroni-corrected *P* value of 0.01 (data not shown), meaning microbial communities were distinct according to habitat type, even when diet treatment was not considered (Fig. 4). In order to determine the roles of diet and tank membership on the microbial community structure, we ran nested ANOSIM tests, with tank nested within diet. Gill and water samples did not show a significant tank or diet effect. Biofilters showed a very strong tank effect, with no effect of fish diet (Table 3 and Fig. 5), suggesting tank membership was a stronger predictor of biofilter microbial communities than fish diet.

**FIG 6:**
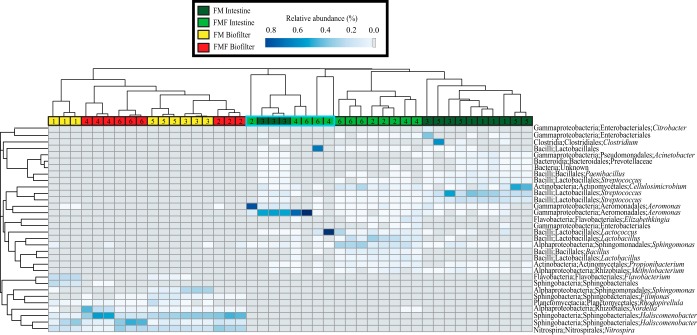
Hierarchical clustering of biofilter and intestine samples across both FM and FMF diets. Each column represents a sample, colored by habitat type and labeled by tank number at the top (note that tanks 1, 3, and 5 are FM while tanks 2, 4, and 6 are FMF). Each row represents the relative abundance of an MED OTU across each sample (including only those OTUs with a minimum of 5% abundance in a single sample). The phylum, order, and genus of each OTU's GAST taxonomy is given at the right; note that multiple distinct OTUs from the same genus are shown. The relative abundance of an MED OTU is depicted using a color scale. The turquoise shading of tank numbers indicates the eight samples containing >50% relative abundance of a single genus, which were removed as outliers for subanalyses of intestinal samples.

Intestine samples showed groupings according to both diet and tank membership (Fig. 4 and 6 and Table 3), although we note these tests are weakly significant and not significant when Bonferroni corrected for multiple ANOSIM tests in this experiment (an alpha of 0.05 after Bonferroni correction for 4 multiple comparisons is 0.0125). However, observation of hierarchical cluster analysis revealed four groupings of intestinal communities, two clusters each composed of similar samples from a given diet, a third cluster composed of samples dominated by Aeromonas, and a final cluster of two highly dissimilar samples each dominated by a single unrelated OTU (Fig. 6). All the samples within these groupings contained a single genus that represents more than 50% of the community, often with a single OTU representing >80% (see above) (Fig. 2 and 6). When these outlier clusters were removed, intestinal habitats formed highly significant clusters according to diet (ANOSIM; *P* = 0.0002) (see Fig. S4 in the supplemental material).

Between-habitat comparisons were not influenced by tank membership. In other words, two habitats in the same tank were as different from each other as the same two habitats between tanks. For example, the mean Bray-Curtis similarity values for water-intestine comparisons within a tank were 1.67 ± 0.3 versus 1.52 ± 0.32 for between-tank comparisons (*P* > 0.1), suggesting intestine samples were no more similar to water samples within their own tank than they were to water in other tanks. We qualify these results, however, by noting that different extraction protocols were used for water and tissue genomic DNAs, potentially leading to differences due to biases of the extraction protocol.

### SIMPER analyses.

To determine what OTUs were driving patterns between habitat types and tanks, we ran SIMPER analyses on Bray-Curtis similarity matrices. Unsurprisingly, SIMPER tests revealed that different OTUs contributed to each habitat's groupings (Table 4), with Sphingobacteriales and Nitrospiraceae contributing to over 25% of biofilter similarities, while OTUs from Lactobacillales, Aeromonadales, and Sphingomonadales contributed to over 30% of intestine habitat similarity (Table 4). These taxa occurred at high abundance in each sample and consistently occurred across all samples. SIMPER analysis also found that groupings of intestinal samples by diet were largely driven by OTUs within the order Lactobacillales (see Table S2 in the supplemental material), a pattern reflected in significant differences of abundant Lactobacillales OTUs across diets (Fig. 3).

**TABLE 4:** Results of SIMPER analysis showing OTUs most characteristic of a given habitat as determined by Bray-Curtis similarity

Classification	% contribution to group similarity
Biofilter (avg similarity, 53.22)	
*Bacteroidetes*; *Sphingobacteria*; *Sphingobacteriales*; *Saprospiraceae*; Haliscomenobacter; OTU 148	11.23
Nitrospirae; Nitrospira; *Nitrospirales*; *Nitrospiraceae*; Nitrospira; OTU 338	7.68
Bacteroidetes; *Sphingobacteria*; *Sphingobacteriales*; *Saprospiraceae*; Haliscomenobacter; OTU 38	4.67
Proteobacteria; Alphaproteobacteria; Rhizobiales; *Phyllobacteriaceae*; *Mesorhizobium*; OTU 7251	2.49
Bacteroidetes; *Sphingobacteria*; *Sphingobacteriales*; Cytophagaceae; Flexibacter; OTU 930	2.43
Bacteroidetes; *Sphingobacteria*; *Sphingobacteriales*; Cytophagaceae; Flexibacter; OTU 937	2.33
Intestine (avg similarity, 25.31)	
Firmicutes; Bacilli; *Lactobacillales*; Streptococcaceae; Streptococcus; OTU 573	12.65
Proteobacteria; Gammaproteobacteria; *Aeromonadales*; *Aeromonadaceae*; Aeromonas; node 3545	8.74
Proteobacteria; Alphaproteobacteria; *Sphingomonadales*; Sphingomonadaceae; Sphingomonas; node 4011	8.41
Actinobacteria; Actinobacteria; Actinomycetales; *Promicromonosporaceae*; *Cellulosimicrobium*; node 5417	8.29
Gill (avg similarity, 26.36)	
Actinobacteria; Actinobacteria; Actinomycetales; *Promicromonosporaceae*; *Cellulosimicrobium*; OTU 5417	15.21
Actinobacteria; Actinobacteria; Actinomycetales; Propionibacteriaceae; Propionibacterium; OTU 104	8.24
Proteobacteria; Gammaproteobacteria; *Enterobacteriales*; Enterobacteriaceae; unknown; OTU 6872	7.84
Water (avg similarity, 35.70)	
Nitrospirae; Nitrospira; *Nitrospirales*; *Nitrospiraceae*; Nitrospira; OTU 338	15.39
Proteobacteria; Betaproteobacteria; *Neisseriales*; Neisseriaceae; unknown; OTU 4013	8.76
Nitrospirae; Nitrospira; *Nitrospirales*; *Nitrospiraceae*; Nitrospira; OTU 337	7.66

## DISCUSSION

### Fish diet does not impact biofilter communities.

Our results provide a deep-sequencing analysis of microbial communities associated with RAS Atlantic salmon production using different diet regimes. We show that alternative feeds do not influence biofilter community composition and demonstrate that several bacterial taxa thought to be involved in nutrient processing remain at high abundance regardless of the fish diet. We also identify several potentially important intestine-associated taxa within the salmon microbiome that are particularly sensitive to diet (Fig. 3; see Table S2 in the supplemental material), confirming previous research with a related fish species (6). Alternative feeds may increase the rates of nutrient excretion by fish (14, 36), potentially changing the water chemistry of the RAS and in turn impacting the critical microbial communities of RAS biofilters. Increased ammonia and nitrate concentrations in water from our FMF treatments corroborate these findings (Table 2), yet we found that the microbial community in biofilters had highly stable community structures and were unaffected by these differences (Fig. 5 and Table 3). Our results show similar biofilter communities across tanks and diets, typified by several Sphingobacteria and Nitrospirales OTUs across all samples in all tanks (Fig. 2 and 6 and Table 4).

Unsurprisingly, many of the dominant taxa found in biofilter habitats play a role in nutrient cycling. Nitrospira, for example, is primarily responsible for oxidation of nitrate in freshwater aquaria (37), and we consistently found several OTUs of the genus at high abundance across all samples. We also found high abundance of Haliscomenobacter OTUs, and although much less is known about this genus, analysis of the full genome sequence from the genus's only species (Haliscomenobacter hydrossis) (38) in the Kyoto Encyclopedia of Genes and Genomes (KEGG) revealed the presence of several denitrifying genes. They include *narB* and *nirBD* (dissimilatory and assimilatory nitrate reduction) and *norBC* and *nosZ* (denitrification), suggesting its role in processing nitrate waste to atmospheric nitrogen. Another taxon found at ubiquitously high abundance across all biofilter samples was Haloferula. Although little is known about the genus, it belongs to the Verrucomicrobia, a phylum of extremely common environmental bacteria representing roughly 7% of cultivatable bacteria in soils from around the globe (39). Furthermore, many Verrucomicrobia are known to fix nitrogen and to live in freshwater systems (40). The most abundant Haloferula OTU in our data set exactly matched a wide range of isolates in GenBank, including both particle-associated and free-living members of the Verrucomicrobia clade CRE-PA, a globally distributed clade in lake and river water (see Fig. 3 in reference 57). Still, the lack of a genome sequence from Haloferula or any member of the CRE-PA clade makes speculation regarding its role in our biofilter habitats difficult.

### Biofilter communities show a strong tank effect.

Interestingly, despite overall stability between samples and diets, we also observed a strong tank effect for biofilter habitats (Fig. 5 and Table 3). A tank effect means samples within an individual RAS (referred to as a tank for simplicity) were more similar to each other than to samples from any other replicate RAS. This effect was driven both by variation in the relative abundances of different OTUs from a common genus (e.g., different Haliscomenobacter OTUs with varying abundances across tanks) and by outlier OTUs that occurred at high abundance in all samples from a given tank but were rare in all others. Several examples typify these outlier OTUs, including a Sphingobacteriales OTU (Haliscomenobacter OTU 5418) found at nearly 3% in all three samples from tank 5 yet extremely rare in all other tanks (Fig. 2). Some outliers occurred in two tanks but no others, including a Flavobacteriales OTU (Flavobacterium OTU 1366) and a Sphingobacteriales OTU (Sphingomonas OTU 5749), which were found at means of 4.5% and 6%, respectively, in all three samples from two tanks yet occurred at less than 0.01% across all others.

Despite these tank-specific differences in individual OTUs, we noted an overall strong similarity and tight clustering of all biofilter habitats (Fig. 2, 4, and 6). The presence of these rare outlier OTUs may be partially explained by stochastic forces previously shown to drive community assembly in other microbial ecosystems (41–43). Stochastic processes of assembly, such as dispersal and random colonization, can lead to differences in habitats with identical abiotic conditions (44) and may be stronger in some habitats than in others. Overall, however, our data are highly suggestive that deterministic processes are overwhelmingly dominant in biofilter communities, as evidenced by strong dominance of Nitrospirales, Sphingobacteriales, and Verrucomicrobiales OTUs across all 18 samples in 6 independent tanks (Fig. 2).

### Intestinal communities differed by diet treatment.

Intestines from both diet treatments were dominated by several OTUs of Lactobacillales, Aeromonadales, and Actinomycetales (Fig. 2 and Table 4; see Table S2 in the supplemental material). These taxa are often found in fish microbiomes across a wide range of species, salinities, and habitats (24, 45–47) and are likely characteristic of fish intestinal microbiota. In 8 of our 26 intestine samples, a single genus represented greater than 50% of the total microbiome, often with only a single OTU representing >80% of the microbiome (Fig. 2). Whether the high abundance of a single OTU represents a disturbed, diseased, or unhealthy state is unknown, although we note that no signs of disease were recorded for any fish collected in this experiment. However, both of the Aeromonas OTUs that dominated six of these communities (OTUs 3545 and 3544) perfectly match several strains of Aeromonas hydrophila, while OTU 3544 also perfectly matches a strain of Aeromonas salmonicida (although this seems unlikely, as A. salmonicida is considered an obligate pathogen and has never been detected on site, either through regular pathogen screening or through clinical observance of furunculosis). Both A. hydrophila and A. salmonicida are economically costly salmon farm pathogens (48), although we note that our data do not permit unambiguous species level taxonomic assignments of any OTU. At any rate, these findings contribute to an understanding that individual carrier fish are likely responsible for the persistence of potential pathogens within fish populations and can act as initial pathogen sources during periods when environmental conditions promote the development of subclinical infections into overt clinical disease outbreaks.

It is possible that shifts in the microbiome resulting from diet were overshadowed by these single, highly abundant OTUs. Observation of hierarchical clustering data and our NMDS plot revealed that the eight intestine samples with a single highly abundant OTU form their own cluster that excludes the remaining samples, which cluster perfectly according to diet (Fig. 6; see Fig. S4 in the supplemental material). Interestingly, when outlier samples were removed from our overall analysis, strongly significant diet-based groupings were observed (Table 3; see Fig. S4 in the supplemental material).

### Changes within the Lactobacillales dominate diet effects.

SIMPER analyses of intestine samples across diets revealed that differences between treatments are driven preeminently by taxa within the order Lactobacillales (see Table S2 in the supplemental material). This pattern is confirmed by significantly greater relative abundance of several Streptococcus OTUs in FM treatments and greater relative abundance of several Lactobacillus OTUs in FMF treatments (Fig. 3), both of which belong to the order Lactobacillales.

Previous research that compared gut microbiomes of salmon fed a grain-based diet versus a fishmeal-based diet found a greater relative abundance of both Lactobacillus and Streptococcus in the grain-fed fish (6), contrary to our finding that Streptococcus is largely replaced by Lactobacillus in fishmeal-free diets. However, both studies underscored the responses of genera within the order Lactobacillales to the absence of fishmeal in salmon diets.

Unfortunately, despite recent efforts to survey the microbiomes of fish using 16S rRNA gene sequence data (49), only a few studies have addressed the functional role of taxa within the microbiome, particularly in relation to fish growth, metabolism, or disease resistance (50). Although our data can demonstrate and corroborate shifts in the microbial community as a function of diet, at this stage, we note no ability to predict how these shifts may impact the physiology of the fish. Nevertheless, the fact that fish are able to perform at similar levels in a farm environment with distinct microbiomes is interesting in the context of host-microbiome interactions, which are often heralded as species specific and of longstanding coevolutionary origin (51–53). Our paper adds to the growing body of literature from a range of host phylogenies that suggests microbiomes can be highly dynamic within a healthy individual, dramatically changing their taxonomic compositions according to environmental factors (24, 41, 54).

Understanding how shifts in intestinal microbial community structure translate into meaningful changes related to fish physiology is a critical next step. Previous work on the roles of alternative feeds, particularly soy, has shown negative impacts of plant-based diets. They include underrepresentation of omega-3 fatty acids (8, 16), an important compound for human health and product marketing. Other studies have shown changes to cell lipid accumulation in liver cells (7), intestinal inflammation (55, 56), and other morphological abnormalities in the intestinal tracts of fish fed alternative diets (19). Morphological or histological examination of the gut was beyond the scope of this study, although we note that mortality and growth rates did not vary between feed treatments in our study (Table 2). Additional studies that test how shifts in microbiome structure may correlate with any abnormality in fish physiology could yield potential probiotic solutions to accompany alternative feeds, alleviating any negative impact associated with shifts in the intestinal microbiome.

### Conclusions.

Biofilters are critical components of recirculating aquaculture systems, with their microbial communities processing nutrients resulting from fish waste and feed decomposition. Assessing the influence of alternative feeds on the ecology of biofilter microbial communities is therefore critical to expanding the use of these feeds across all water recirculation systems. Despite some variation in water chemistry (e.g., ammonium/nitrate) attributable to treatment, we found that biofilter microbial communities are not impacted by fish diet. We also corroborated a previous finding that salmon intestinal communities varied with diet treatments but showed that these changes occurred between closely related microbial taxa and did not impact fish performance. Our study provides support for the hypothesis that novel protein diets are viable alternatives to traditional fishmeal-based diets in water recirculation systems. Finally, we note that additional work is needed to better understand the function of particular microbial taxa in fish physiology so that changes to the microbial community determined by studies such as ours can be informative in the management, performance, and profitability of aquaculture operations.

## Supplementary Material

Supplemental material
