# Supplementary material for: Influence of Fishmeal-Free Diets on Microbial Communities in Atlantic Salmon (Salmo salar) Recirculation Aquaculture Systems
Source: Appl Environ Microbiol. 2016 Jul 15;82(15):4470–81. doi: 10.1128/AEM.00902-16 (PMC4984271; doi:10.1128/AEM.00902-16)
Supplement: Supplemental material [file supp_82_15_4470__index.html]

Influence of Fishmeal-Free Diets on Microbial Communities in Atlantic Salmon (Salmo salar) Recirculation Aquaculture Systems — Supplemental material 

# Influence of Fishmeal-Free Diets on Microbial Communities in Atlantic Salmon (Salmo salar) Recirculation Aquaculture Systems

## Supplemental material

- Supplemental file 1 -

  Mean (±SE) alpha diversity metrics for all sample types in this study based on MED OTUs subsampled to 25,000 sequences (Fig. S1); mean relative abundance of the most abundant 10 OTUs from a given habitat (Fig. S2); hierarchical clustering of all samples and all OTUs across both FM and FMF diets (Fig. S3); NMDS plot of microbial communities from intestine samples (Fig. S4); water quality parameters evaluated and descriptions of methodologies and frequency of testing for each (Table S1); SIMPER analysis of distinguishing OTUs between FM and FMF diets in intestinal samples (Table S2).

  PDF, 816K
- Supplemental file 2 -

  List of DNA sequences identified as contaminants according to the methods section and removed from analysis prior to OTU clustering (Data Set S1).

  XLSX, 39K
- Supplemental file 3 -

  MIMARKS-compliant data for each sample (Data Set S2).

  XLS, 114K
- Supplemental file 4 -

  Raw data values for water chemistry (Data Set S3).

  XLSX, 122K
