# Supplementary material for: Influence of Fishmeal-Free Diets on Microbial Communities in Atlantic Salmon (Salmo salar) Recirculation Aquaculture Systems
Source: Appl Environ Microbiol. 2016 Jul 15;82(15):4470–81. doi: 10.1128/AEM.00902-16 (PMC4984271; doi:10.1128/AEM.00902-16)
Supplement: Supplemental material [file AEM.00902-16_zam999117249so1.pdf]

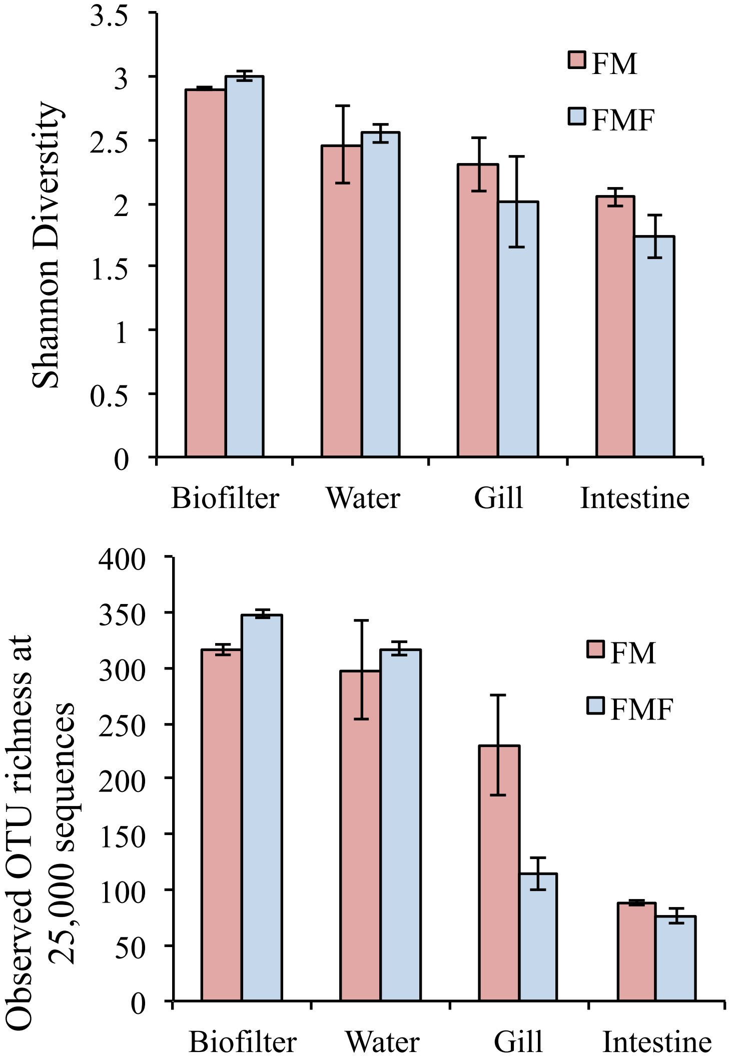

Figure S1: Mean (±SE) alpha diversity metrics for all sample types in this study based on MED OTUs subsampled to 25,000 sequences. Only biofilter samples show significant differences between Fishmeal (FM) and Fishmeal Free (FMF) diets.

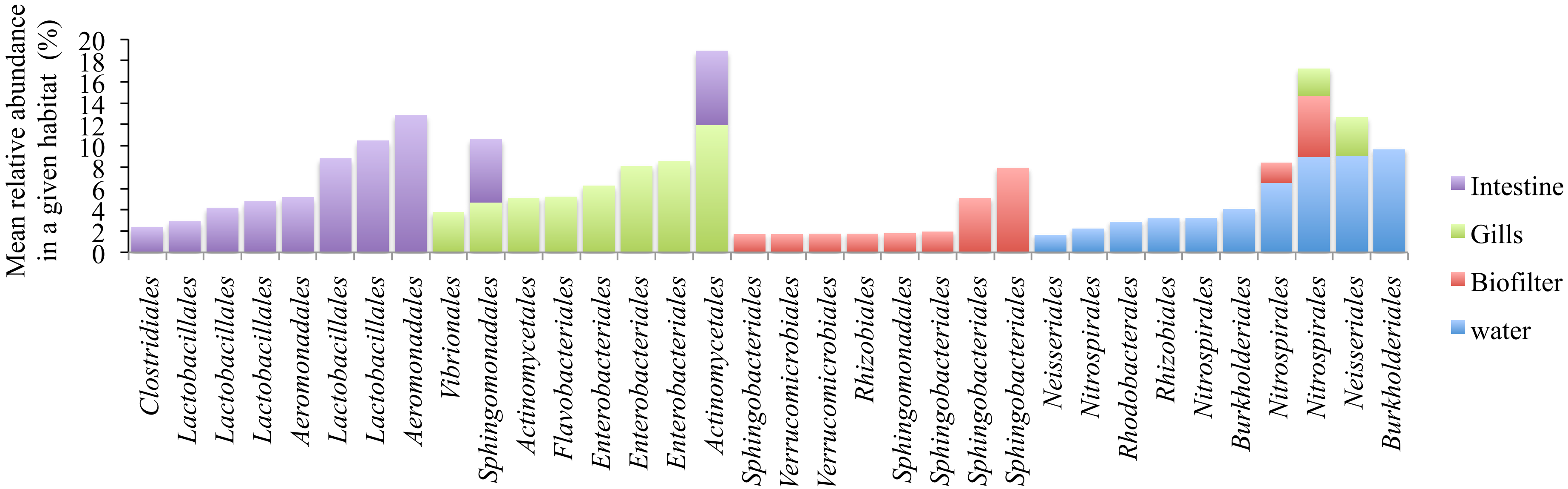

Figure S2: Mean relative abundance of the most abundant 10 OTUs from a given habitat are shown, and colored according to the habitat in which they are most abundant. OTUs are labeled by order. Note that OTUs with the same order label (e.g. multiple *Lactobacillales*) are distinct OTUs within that order. Note that only a single OTU is found in more than 2 habitats.

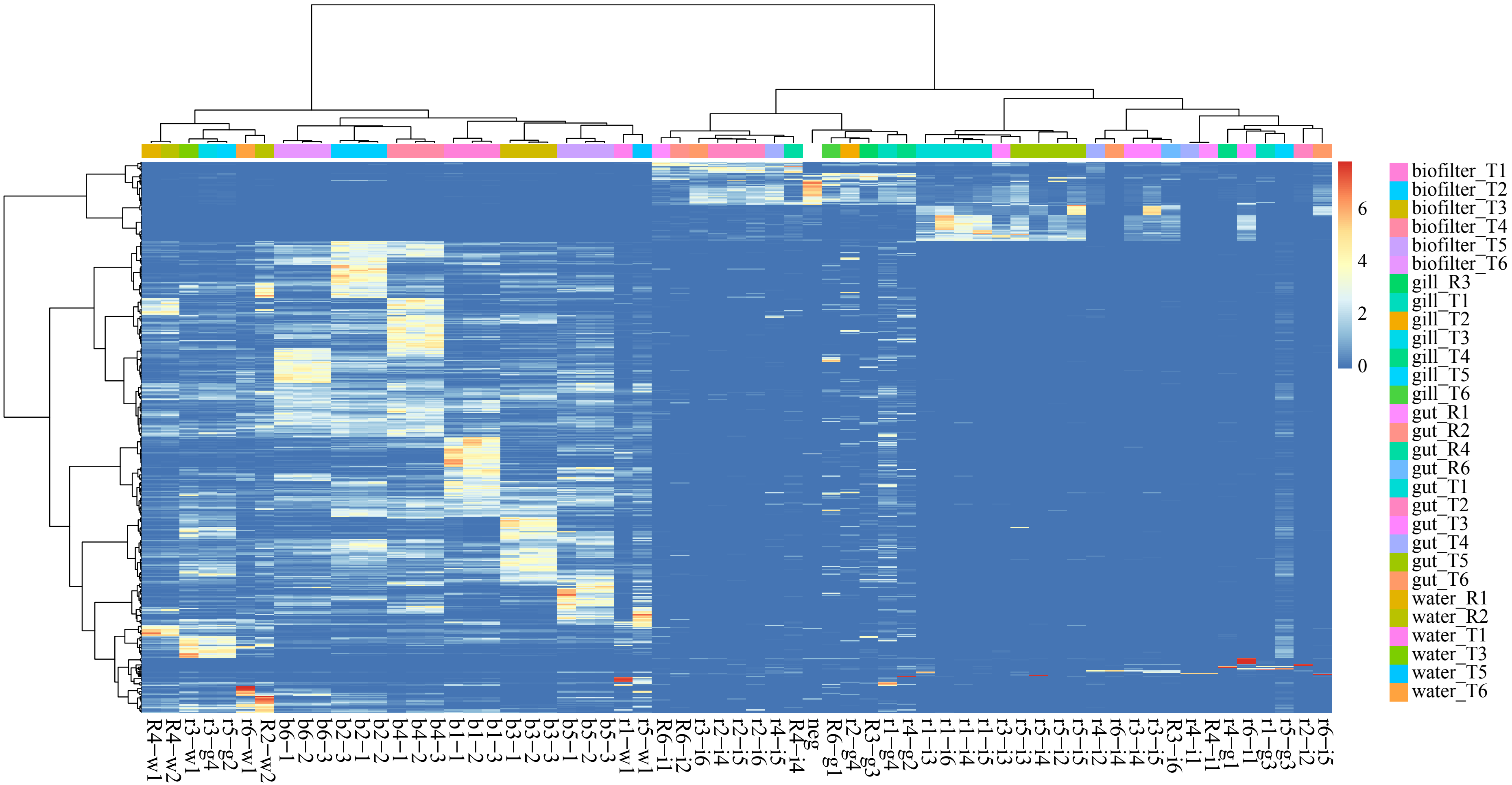

Figure S3: Hierarchical clustering of all samples and all OTUs across both FM and FMF diets. Each column represents a sample, colored by sample type, while each row represents an MED OTU. Relative abundance of an MED OTU is depicted using a color scale. Because so many OTUs are shown, they are not labeled.

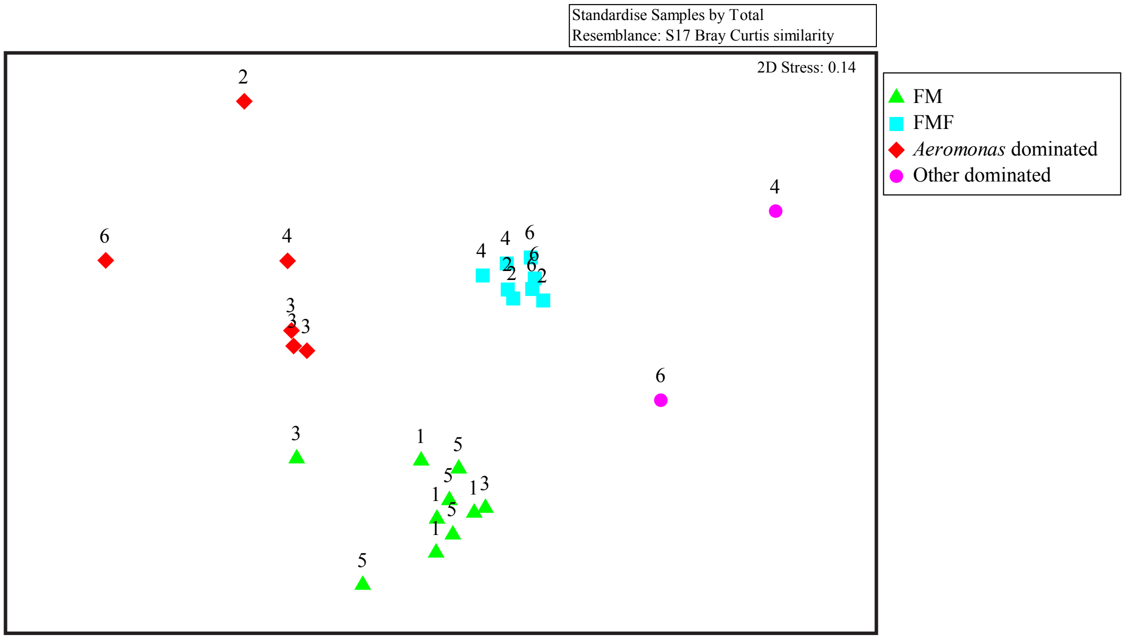

Figure S4: Non-Metric Dimensional Scaling (NMDS) plot of microbial communities from intestine samples only. Samples are colored according to diet (FM = Fishmeal, FMF = Fishmeal free), or if a given sample had greater than 50% relative abundance represented by a single genus, it is considered an outlier and colored according to that genus. ANOSIM tests shows highly significant groupings according to diet when outlier samples are removed from analyses. Tank membership for each sample is also shown.

Table S1. Water quality parameters evaluated and descriptions of methodologies and frequency of testing for each.

Parameter	Method of Analysis	Data Collection Frequency
Alkalinity	Standard Methods 2320 - Sulfuric Acid Titration	Twice weekly
Carbon Dioxide	Hach Method 8223 - Buret Titration	Once weekly
$CBOD_5$	Standard Methods 5210B - 5 day test (no sample prefiltration)	Once weekly
Dissolved Oxygen	Hach SC100 Universal Controller & LDO® Probe	Daily
Heterotrophic Bacteria Count	Standard Methods 9215D - Membrane Filtration and Agar Plate Counts	Once weekly
Nitrate Nitrogen	Hach Method 8171 - Cadmium Reduction	Once weekly
Nitrite Nitrogen	Hach Method 8507 - Diazotization	Once weekly
Oxidative Reduction Potential	Hach SC100 Universal Controller & Differential ORP Sensor	Daily
pH	Hach Method 8156 – pH electrode	Twice weekly
Temperature	Hach SC100 Universal Controller & Differential ORP Sensor	Daily
Total Ammonia Nitrogen	Hach Method 8038 - Nessler	Once weekly
Total Nitrogen	Hach Methods 10071, 10072 – Persulfate Digestion Method	Once weekly
Total Phosphorus	Hach Method 8190 - Acid Persulfate Digestion	Once weekly
Total Suspended Solids	Standard Methods 2540D - Dried at 103-105 ^{o}C	Once weekly
True Color	Hach Method 8025 - Platinum-Cobalt	Once weekly
Ultraviolet Transmittance	Standard Methods 5910B - Ultraviolet Absorption	Once weekly

Table S2: SIMPER analysis of distinguishing OTUs between FM and FMF diets in intestinal samples, after outlier samples were removed. Table shows average relative abundance in each treatment, and the relative contribution of each OTU to the Bray-Curtis dissimilarity metric.

Species	Av.Abund FM	Av.Abund FMF	Contrib%
Bacteria;Firmicutes;Bacilli;Lactobacillales;Streptococcaceae;Streptococcus; OTU573	20.1	1.9	11.45
*Bacteria;Proteobacteria;Alphaproteobacteria;Sphingomonadales;Sphingomonadaceae;Sphingomonas;*OTU4011	2.15	15.39	8.38
Bacteria;Firmicutes;Bacilli;Lactobacillales;Lactobacillaceae;Lactobacillus; OTU684	0.13	12.93	8.05
Bacteria;Actinobacteria;Actinobacteria;Actinomycetales;Promicromonosporaceae;Cellulosimicrobium;OTU5417	9.81	8.78	7.34
Bacteria;Firmicutes;Bacilli;Lactobacillales;Streptococcaceae;Streptococcus; OTU576	10.93	1.2	6.12
Bacteria;Firmicutes;Bacilli;Lactobacillales;Streptococcaceae;Lactococcus; OTU107	1.61	7.37	4.24
*Bacteria;Firmicutes;Clostridia;Clostridiales;Clostridiaceae;Clostridium;*OTU7757	6.22	0	3.91
Bacteria;Firmicutes;Bacilli;Bacillales;Bacillaceae;Bacillus; OTU3198	0.03	5.51	3.44
Bacteria;Firmicutes;Bacilli;Lactobacillales;Lactobacillaceae;Lactobacillus; OTU8245	0.04	4.61	2.88
